# Successful Surgical Management of Ameloblastic Fibro‐Odontoma in the Posterior Maxilla: A Rare Case Report

**DOI:** 10.1002/ccr3.72013

**Published:** 2026-02-08

**Authors:** Saman Abbasi, Maryam Mohebiniya, Salar Nasr Esfahani

**Affiliations:** ^1^ Department of Oral and Maxillofacial Surgery, School of Dentistry Arak University of Medical Sciences Arak Iran; ^2^ Department of Oral and Maxillofacial Radiology, School of Dentistry Arak University of Medical Sciences Arak Iran; ^3^ Department of Pathology, School of Medicine Arak University of Medical Sciences Arak Iran

**Keywords:** maxillary neoplasms, odontogenic tumors, odontoma, oral surgery

## Abstract

Ameloblastic fibro‐odontoma (AFO) is a rare benign mixed odontogenic tumor that exhibits histological features of both ameloblastic fibroma and complex odontoma. This report presents a rare case of AFO in a 12‐year‐old male, located in the posterior maxilla—an uncommon site for its occurrence.

## 1 Introduction

Ameloblastic fibro‐odontoma (AFO) is a rare, benign, slow‐growing mixed odontogenic tumor composed of odontogenic epithelium and ectomesenchymal cellular tissue [1]. Initially classified as a distinct entity by Hooker in 1972, the 2017 World Health Organization (WHO) classification redefined AFO as a “developing odontoma” rather than a separate tumor [2]. AFO accounts for approximately 1%–3% of all odontogenic tumors [3]. It most commonly occurs in the posterior mandible, while its presence in the maxilla is extremely rare. The average age of occurrence is reported to be 11.5 years [4].

Clinically, AFO typically presents as a painless swelling and is often associated with delayed tooth eruption [3]. Histologically, it shares features with ameloblastic fibroma (AF) but is distinguished by the presence of hard dental tissues [5]. Radiographically, an AFO appears as a well‐demarcated radiolucent lesion containing radiopaque areas, frequently associated with an impacted tooth [1, 6]. Although it is generally noninvasive, recurrence is rare according to most literature. The standard treatment is conservative, involving enucleation and curettage [4].

This report presents a rare case of an expansile AFO affecting the left maxilla and extending into the maxillary sinus. The case highlights its clinical, radiographic, and histopathological characteristics, as well as the surgical approach and outcomes.

## 2 Case History/Examination

**FIGURE 1:**
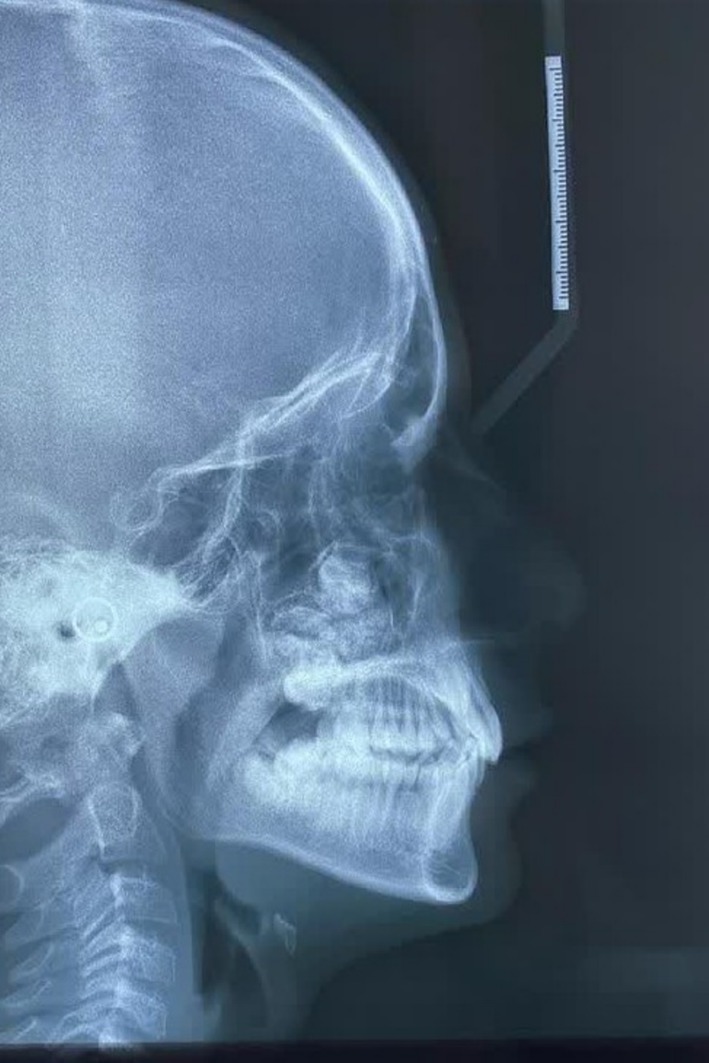
Lateral cephalometric radiograph showing a well‐defined mixed radiolucent–radiopaque lesion in the posterior maxilla.

A 12‐year‐old male patient was referred for a dental evaluation due to tooth crowding in preparation for orthodontic treatment. During the initial examination, a mixed lesion was incidentally detected in the maxillary region on lateral radiography (Figure 1). Given the nature of the finding, the dentist referred the patient to an oral and maxillofacial surgeon for further assessment. To obtain a comprehensive evaluation, the surgeon requested a cone beam computed tomography (CBCT) scan of the posterior left maxilla.

The patient's medical history was unremarkable, and an extraoral examination revealed no facial asymmetry. The patient was asymptomatic, reporting no pain or discomfort. Intraoral examination revealed a mild, localized swelling in the buccal aspect of the left posterior maxilla, while the overlying gingival tissue appeared normal.

**FIGURE 2:**
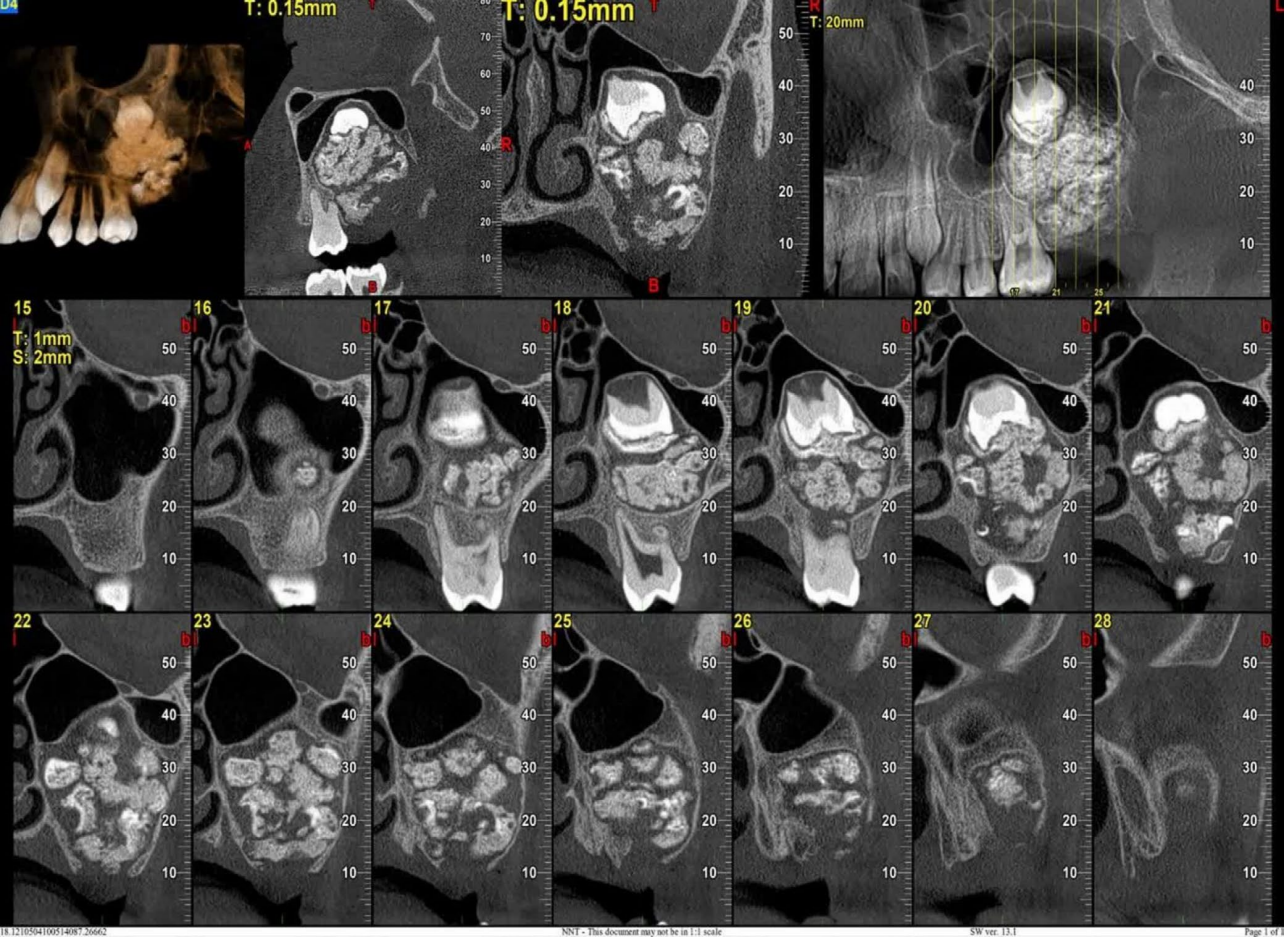
CBCT analysis using reformatted panoramic, sagittal, and 3D transparent views reveals a well‐circumscribed, mixed pericoronal lesion in the posterior region of the left maxilla.

**FIGURE 3:**
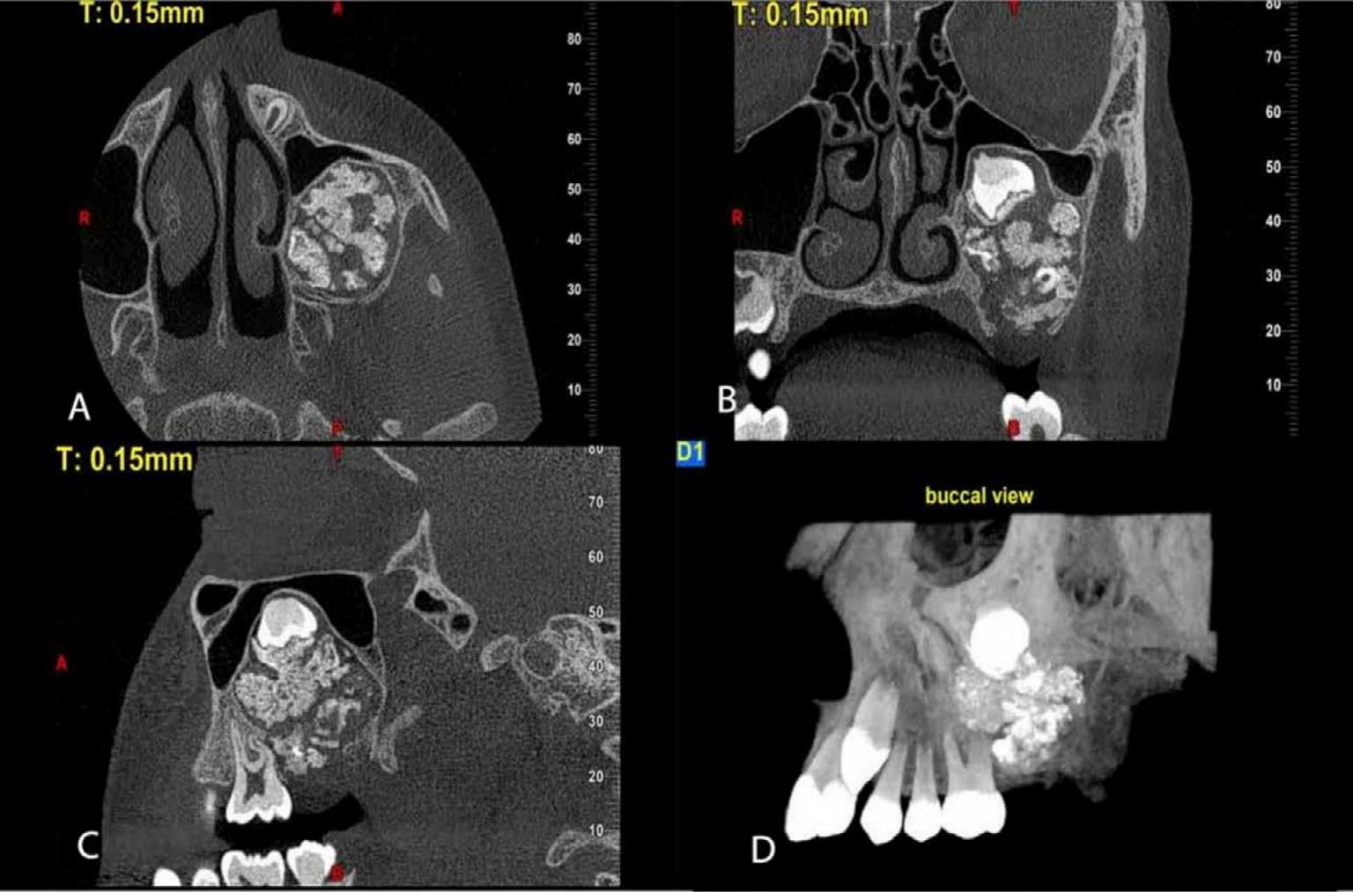
Preoperative CBCT. (A) axial view, (B) coronal view, (C) sagittal view, (D) reconstructed view (MIP).

CBCT imaging was acquired and analyzed in axi, sagittal, and cross‐sectional planes, as well as three‐dimensional reconstructions (Figures 2 and 3). The scans revealed a well‐defined, corticated expansile lesion with mixed attenuation in the posterior left maxilla, located within the alveolar bone and pericoronally associated with an impacted second molar. The lesion measured 34 mm in vertical height, 25 mm in buccopalatal width, and 26 mm in anteroposterior length, and was surrounded by a radiolucent rim. Internally, the lesion exhibited a heterogeneous appearance, containing both radiolucent and radiopaque components. Multiple tooth‐like radiopaque structures were identified, demonstrating densities comparable to enamel and dentin. The lesion caused buccal and palatal cortical expansion, with thinning of the cortical plates and involvement of the inferior and medial walls of the left maxillary sinus. Additionally, the floor of the left maxillary sinus was elevated, leading to a marked reduction in sinus volume. However, the left maxillary sinus ostium remained patent, and no mucosal thickening was observed in the maxillary sinus space.

Further analysis showed perforation of the alveolar crest in the distal region of the left first molar. While the lamina dura and periodontal ligament space were indistinct at the distal root of the left first molar, no evidence of root resorption was observed.

## 3 Differential Diagnosis, Investigations, and Treatment

Based on clinical and radiographic findings, differential diagnoses included complex‐compound odontoma, AFO, and calcifying cystic odontogenic tumor. After a thorough evaluation of the CBCT images, the oral and maxillofacial surgeon determined that lesion removal was necessary. In this case, surgical treatment was selected due to the lesion's considerable size, risk of continued expansion into the maxillary sinus, and close association with an unerupted molar. Despite the lack of symptoms, CBCT imaging showed cortical thinning and sinus floor elevation, suggesting potential future complications if left untreated. Therefore, early intervention was deemed necessary to prevent structural damage and allow for conservative enucleation while the lesion remained well‐circumscribed.

**FIGURE 4:**
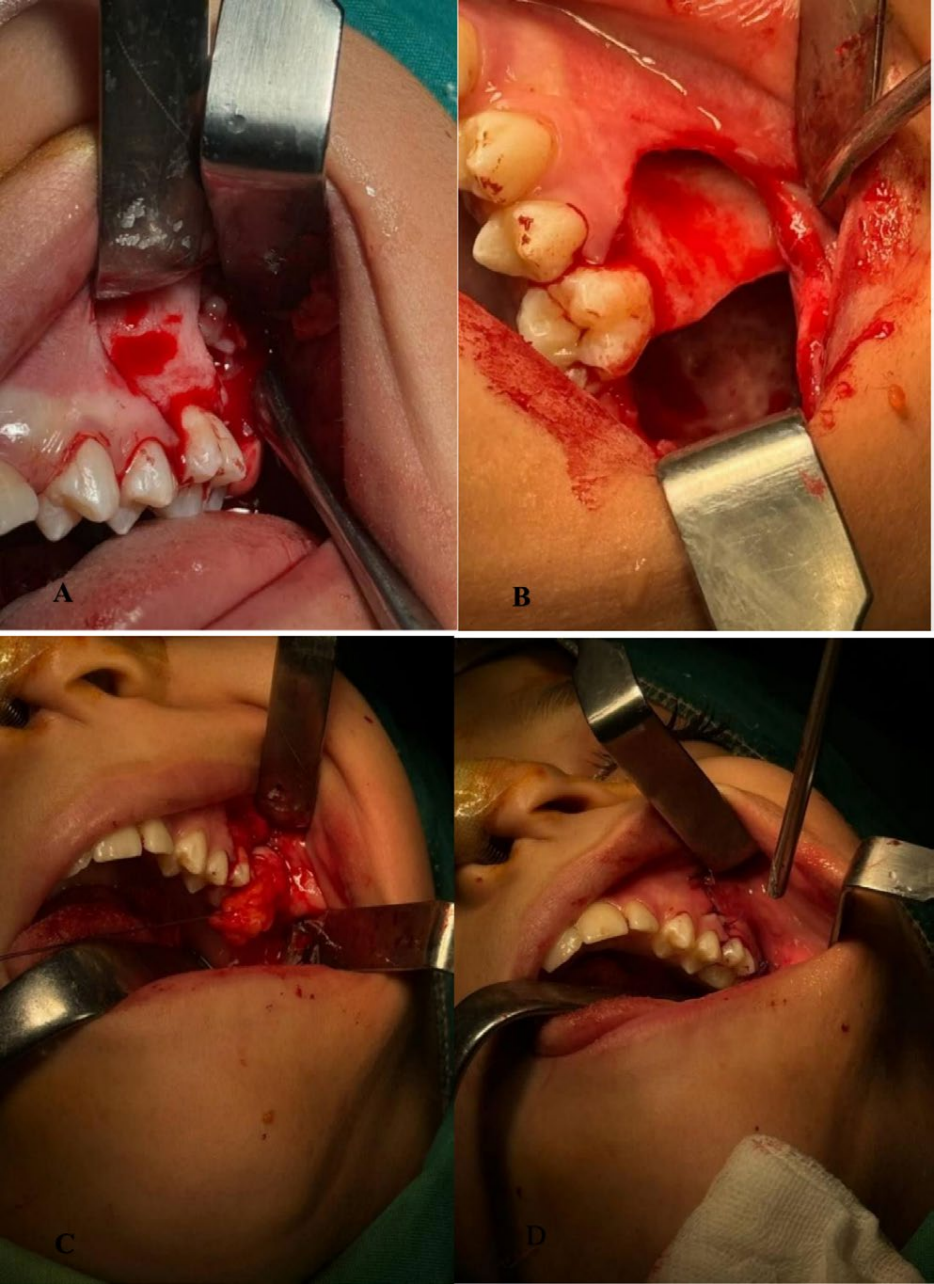
Intraoperative views of the surgical approach. (A, B) Surgical enucleation and curettage of the lesion via an intraoral approach to the left posterior maxilla. (C, D) Release and advancement of the buccal fat pad to facilitate wound closure and achieve a tension‐free suture.

Routine hematological investigations were found to be normal. The procedure was performed under general anesthesia with an intraoral approach approximately 10 days after the initial CT scan. A full‐thickness triangular mucoperiosteal flap was carefully elevated to ensure adequate access to the lesion. Upon exposure, Enucleation, curettage, and extraction of the impacted tooth were performed to completely remove the lesion, followed by osteotomy and meticulous removal of any residual bone fragments using a carbide bur (Figure 4).

To facilitate optimal wound closure, the buccal fat pad was mobilized and positioned securely against the palatal gingiva. The buccal flap was then repositioned over the fat pad to ensure stability. Given the size of the surgical cavity, the buccal fat pad was chosen to enhance healing and reduce the risk of wound dehiscence (Figure 4). The procedure was concluded with primary closure using Vicryl 3–0 sutures (Figure 4).

**FIGURE 5:**
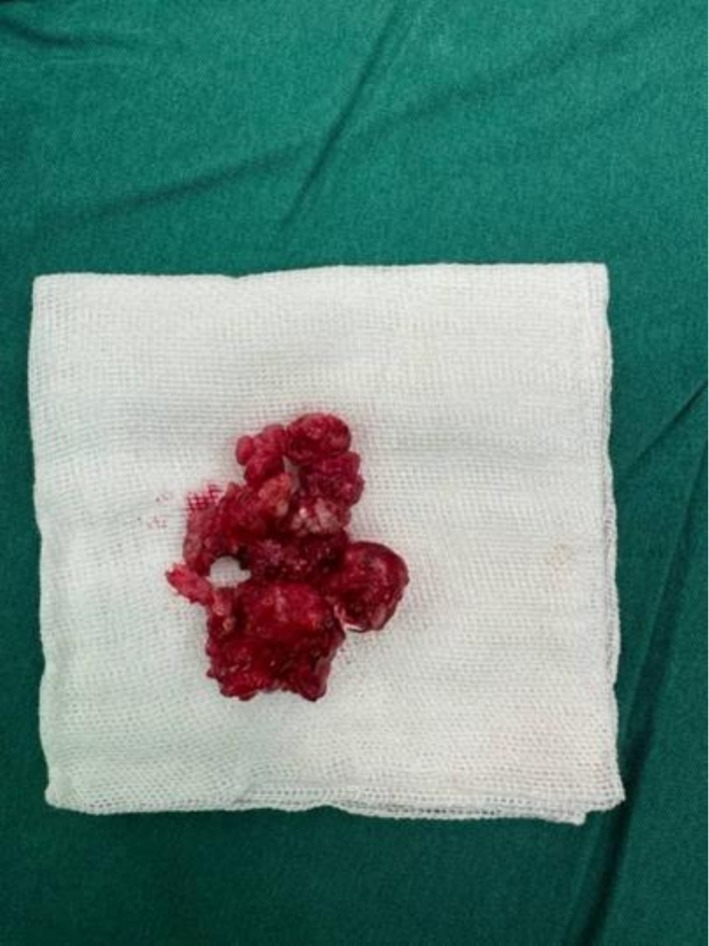
Gross specimen composed of calcified tissue and multiple denticles, consistent with a mixed odontogenic tumor.

**FIGURE 6:**
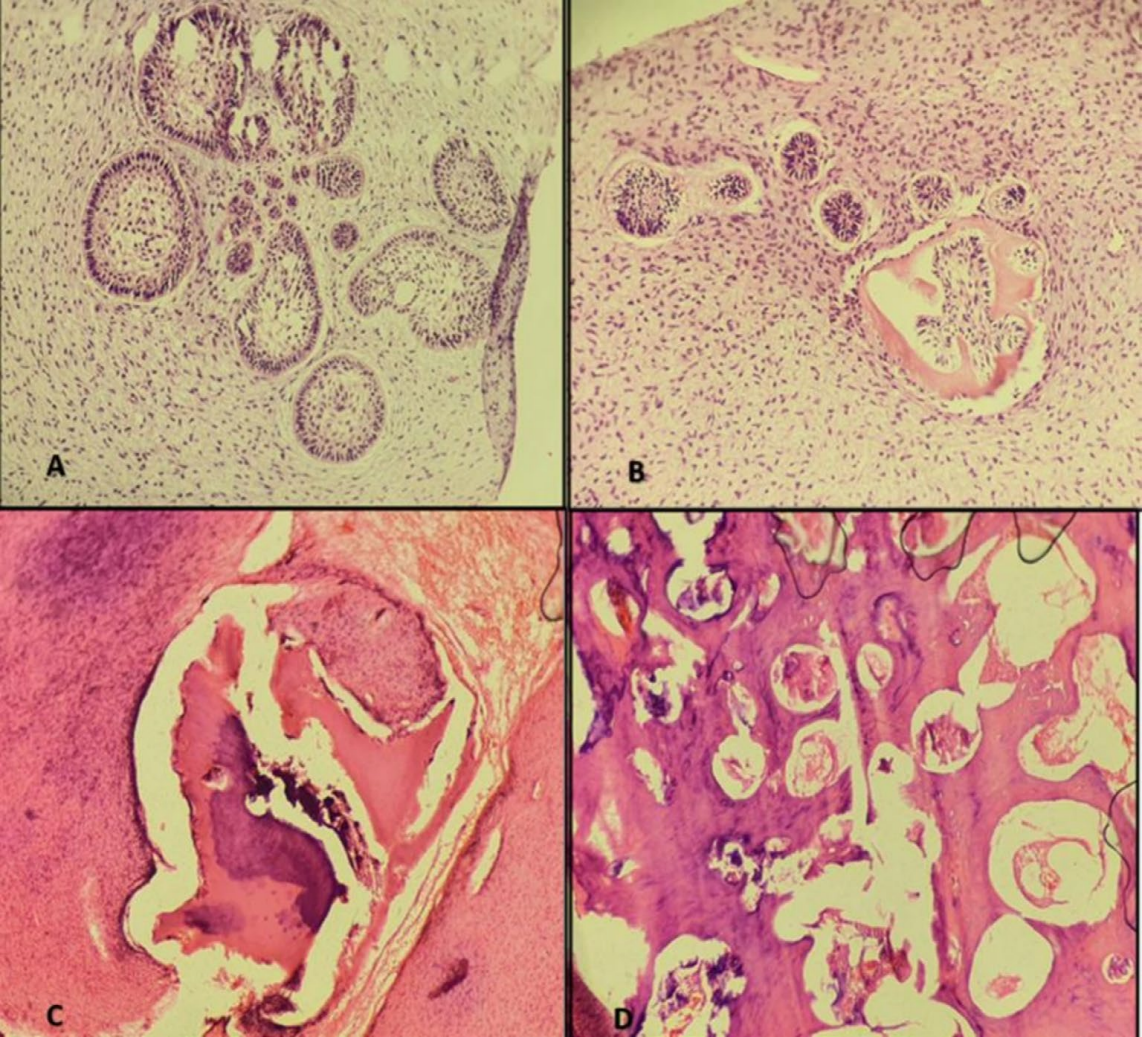
(A, B) Histological sections of soft tissue showing small islands and cords of attenuated odontogenic (ameloblastic) epithelium, typically two cells thick, within a dense, immature collagenous stroma. Stellate reticulum–like areas and initial cementum formation are also observed. (C, D) Hard tissue sections reveal irregularly arranged enamel and dentin masses in close association with ameloblastic epithelium.

The excised specimen was immediately fixed in formalin and sent for histopathological examination (Figure 5). Microscopic evaluation revealed small islands and cords of markedly attenuated odontogenic (ameloblastic) epithelium, typically two cells thick, embedded within a dense, immature collagenous stroma. Stellate reticulum–like areas were also noted, along with initial cementum formation in some regions. Hard tissue sections showed irregular masses of enamel and dentin arranged in a disorganized pattern, closely associated with ameloblastic epithelium. No features suggestive of malignancy were identified. These findings confirmed the diagnosis of AFO (Figure 6).

Postoperatively, the patient was prescribed analgesics, antibiotics, and chlorhexidine mouthwash to support healing and prevent infection.

## 4 Conclusion and Results (Outcome and Follow‐Up)

**FIGURE 7:**
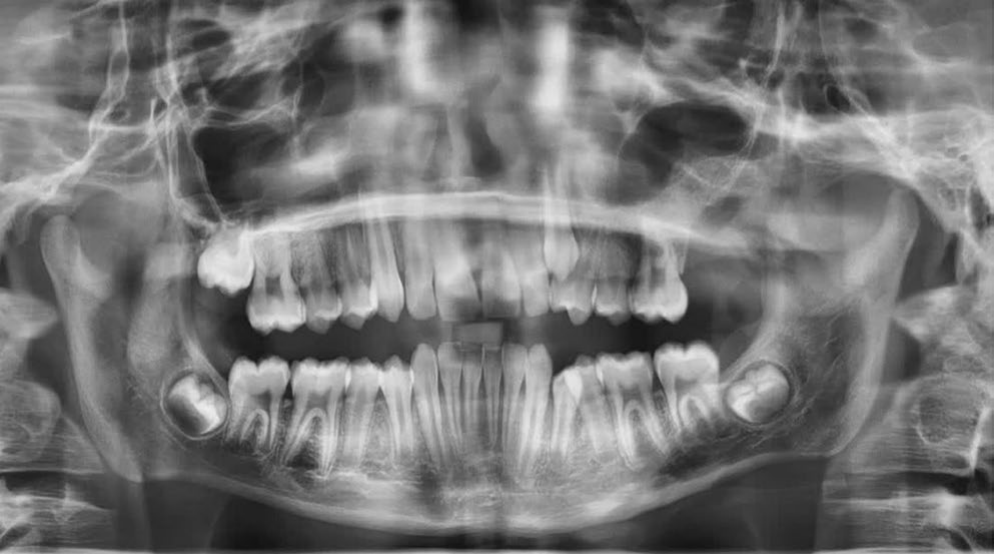
Panoramic radiograph taken at 5‐month follow‐up, demonstrating complete healing of the surgical site with no evidence of recurrence.

Serial follow‐up evaluations at 1 week, 1 month, and 3 months postoperatively revealed an uneventful recovery, with no clinical signs of complications. The patient remained asymptomatic and reported no functional limitations during this period. A panoramic radiograph obtained at the 5‐month follow‐up confirmed complete osseous healing at the surgical site, with no evidence of residual lesion or recurrence. The internal space of the left maxillary sinus appeared clear, with no signs of inflammation or infection (Figure 7).

No oroantral fistula developed during the postoperative course. The patient reported no pain, infection, sinusitis, or discomfort, and resumed normal masticatory and oral function within 10 days. No additional medications were required beyond the initially prescribed analgesics and antibiotics. Overall recovery was excellent, and the patient expressed full satisfaction with the treatment outcome.

Although the current follow‐up duration is limited, we acknowledge this as a study limitation. Long‐term clinical and radiographic surveillance is ongoing, and the patient will continue to be monitored for at least 1 year. These findings are consistent with the generally favorable prognosis of AFO following complete surgical excision, though extended observation is recommended to detect any rare instances of delayed recurrence.

This study presents a rare case of AFO in the posterior maxilla, an uncommon location for such lesions. The lesion was incidentally detected during a routine dental and radiographic examination. This case underscores the critical importance of regular dental examinations and radiographic assessments, as many lesions, including AFO, often remain asymptomatic and are typically discovered incidentally. Clinicians should consider AFO in the differential diagnosis when encountering a mixed radiolucent‐radiopaque mass in the maxilla associated with an unerupted tooth. Surgical excision remains the treatment of choice. Although AFO is generally benign, minimally invasive, and has a very low recurrence rate, long‐term follow‐up is recommended to monitor for potential malignant transformation. This report emphasizes the importance of early detection, thorough radiologic evaluation, and timely surgical intervention in the effective management of rare odontogenic tumors.

## 5 Discussion

Ameloblastic fibro‐odontoma is a rare, benign, mixed odontogenic tumor composed of both epithelial and mesenchymal components. First described by Hooker in 1972 as a distinct odontogenic entity [7], AFO was historically classified separately from odontoma. However, in the 2017 WHO classification, it was redefined as a “developing odontoma” and grouped under the odontoma category [2]. This reclassification is based on the hypothesis that AFO represents an intermediate stage in the maturation of odontogenic tissue, eventually progressing into a complex or compound odontoma [8]. Despite broad acceptance of this theory, some reports continue to describe AFO as a separate neoplastic entity, particularly in cases demonstrating aggressive behavior or atypical histopathological features [4].

Odontogenic tumors account for approximately 7% of all oral lesions in children and adolescents, with AFO comprising about 1%–3% of these lesions [3, 9]. Studies indicate a male predilection, with the majority of cases occurring in patients under 20 years of age. AFO most commonly affects the posterior mandible (approximately 60%) and is frequently associated with an impacted tooth [4]. In contrast, cases involving the posterior maxilla are relatively rare [4, 8, 10]. Due to its slow growth, AFO is often asymptomatic and may present as a painless swelling or delayed tooth eruption. In many cases, including ours, it is discovered incidentally during routine radiographic imaging for unrelated dental issues.

The current case involved a 12‐year‐old male with an asymptomatic lesion in the posterior maxilla, identified during an orthodontic evaluation. This rare presentation highlights the importance of regular radiographic monitoring in pediatric patients, even in the absence of clinical symptoms.

Radiographically, an AFO typically presents as a well‐circumscribed, unilocular radiolucent lesion containing variable amounts of radiopaque material, often associated with the crown of an unerupted tooth [11]. The quantity and appearance of the radiopaque component can vary widely; in some cases, it may be extensive enough to resemble an odontoma [12].

The standard management of AFO is conservative. Most lesions are successfully treated through enucleation or curettage, owing to their well‐defined margins and minimal invasiveness, which allow for clean separation from surrounding bone [4, 13]. Recurrence is rare—approximately 5.5%—and is usually attributed to incomplete surgical removal [9]. For example, in Tsagaris' review of 29 cases, only one recurrence occurred, which was linked to inadequate initial excision [9]. Malignant transformation is extremely rare and has primarily been reported in adult patients [4, 8].

The management of associated impacted teeth remains a matter of debate. Some authors advocate for preserving unerupted teeth when possible [14, 15], while others recommend extraction to reduce the risk of recurrence [4]. In cases where histological findings show disorganized fibrous stroma or a loss of odontogenic epithelium, more aggressive surgical intervention may be necessary. Nivin Omar et al. reported a case involving significant bone destruction in the maxilla, which required hemimaxillectomy [4].

Compared to mandibular cases, maxillary AFOs may pose greater surgical challenges due to proximity to the sinus, thinner cortical bone, and potential risk of oroantral communication. As demonstrated in our case, early intervention enabled complete excision and sinus preservation [16].

In the present case, the patient had no complaints and presented solely for orthodontic evaluation, unlike most previously reported cases involving the posterior maxilla, where symptoms such as swelling were common [4, 8]. Notably, the patient had never undergone routine dental checkups, which allowed the lesion to grow undetected and expand into the maxillary sinus without the awareness of the patient or family. Given the lesion's considerable size and its association with an unerupted second molar, surgical enucleation combined with removal of the impacted tooth was performed to reduce the risk of recurrence. At the six‐month follow‐up, the surgical site showed satisfactory healing with no signs of complications or recurrence.

Few cases of AFO involving the posterior maxilla have been reported in the literature. For example, Baker and Swift (1993) described a case located in the anterior maxilla, while Jahanshahi Afshar et al. (2022) presented a posterior maxillary case in a 4‐year‐old boy with evident facial asymmetry and intraoral swelling [8, 10]. In contrast, our patient was entirely asymptomatic, and the lesion was discovered incidentally during routine orthodontic evaluation. Additionally, Nivin Omar et al. (2021) reported a large expansile AFO measuring 5.5 × 4.3 cm in a 21‐year‐old male, which required an aggressive surgical approach, including hemimaxillectomy and partial palatectomy, due to extensive bone destruction and rapid lesion growth [4]. Compared to these cases, our patient was successfully treated with conservative surgical enucleation and extraction, and complete healing was observed during follow‐up. This comparison highlights the broad clinical spectrum of AFO and emphasizes the need for individualized treatment planning based on lesion size, location, radiographic features, and clinical behavior. Despite the variation in treatment modalities, no recurrence was observed in the previously reported maxillary cases during their respective follow‐up periods [4, 8, 10].

This case underscores the importance of annual clinical and radiographic examinations in children and adolescents, not only for caries detection but also to evaluate potential causes of tooth impaction. Many odontogenic cysts and tumors, such as AFO, can remain asymptomatic for long periods. Early detection allows for timely intervention, which may prevent lesion enlargement and associated complications, enabling more conservative and successful management.

## Author Contributions

**Saman Abbasi:** conceptualization, investigation, supervision. **Maryam Mohebiniya:** data curation, investigation, project administration, writing – original draft, writing – review and editing. **Salar Nasr Esfahani:** investigation, validation, writing – review and editing.

## Funding

The authors have nothing to report.

## Consent

Written informed consent was obtained from the patient to publish this report in accordance with the journal's patient consent policy.

## Data Availability

Data sharing not applicable to this article as no datasets were generated or analyzed during the current study.
